# A 3D Chemically Modified Graphene Hydrogel for Fast, Highly Sensitive, and Selective Gas Sensor

**DOI:** 10.1002/advs.201600319

**Published:** 2016-12-20

**Authors:** Jin Wu, Kai Tao, Yuanyuan Guo, Zhong Li, Xiaotian Wang, Zhongzhen Luo, Shuanglong Feng, Chunlei Du, Di Chen, Jianmin Miao, Leslie K. Norford

**Affiliations:** ^1^ School of Mechanical and Aerospace EngineeringNanyang Technological UniversitySingapore639798Singapore; ^2^ Micro‐Nano Manufacturing and System Integration CenterChongqing Institute of Green and Intelligent TechnologyChinese Academy of SciencesChongqing400714P. R. China; ^3^ School of Materials Science and EngineeringNanyang Technological University50 Nanyang AvenueSingapore639798Singapore; ^4^ Key Laboratory for Thin Film and Microfabrication Technology of Ministry of EducationDepartment of Instrument Science and EngineeringSchool of Electronic Information and Electrical EngineeringShanghai Jiao Tong University800 Dongchuan RoadShanghai200240P. R. China; ^5^ Shanghai Engineering Research Center for Intelligent Diagnosis and Treatment Instrument800 Dongchuan RoadShanghai200240P. R. China; ^6^ Center for Environmental Sensing and Modeling (CENSAM)Singapore‐MIT Alliance for Research and Technology (SMART) CentreSingapore117543Singapore; ^7^ Department of ArchitectureMassachusetts Institute of TechnologyCambridgeMA02139USA

**Keywords:** 3D reduced graphene oxide hydrogel, chemical modification, gas sensor, microheater, sulfonated

## Abstract

Reduced graphene oxide (RGO) has proved to be a promising candidate in high‐performance gas sensing in ambient conditions. However, trace detection of different kinds of gases with simultaneously high sensitivity and selectivity is challenging. Here, a chemiresistor‐type sensor based on 3D sulfonated RGO hydrogel (S‐RGOH) is reported, which can detect a variety of important gases with high sensitivity, boosted selectivity, fast response, and good reversibility. The NaHSO_3_ functionalized RGOH displays remarkable 118.6 and 58.9 times higher responses to NO_2_ and NH_3_, respectively, compared with its unmodified RGOH counterpart. In addition, the S‐RGOH sensor is highly responsive to volatile organic compounds. More importantly, the characteristic patterns on the linearly fitted response–temperature curves are employed to distinguish various gases for the first time. The temperature of the sensor is elevated rapidly by an imbedded microheater with little power consumption. The 3D S‐RGOH is characterized and the sensing mechanisms are proposed. This work gains new insights into boosting the sensitivity of detecting various gases by combining chemical modification and 3D structural engineering of RGO, and improving the selectivity of gas sensing by employing temperature dependent response characteristics of RGO for different gases.

## 1 Introduction

The production and emission of toxic gases in industry and agriculture increasingly endanger the health of human beings in the long term.^1^, ^2^, ^3^, ^4^ For example, NO_2_ gas acts as a source of acid rain and contributes to the formation of ozone (O_3_), which is the major cause of photochemical smog.^5^, ^6^ NO_2_ at a concentration higher than 1 ppm can also cause serious diseases to people's respiratory system.^2^, ^6^, ^7^ Furthermore, ammonia (NH_3_) is a toxic and colorless gas with permissible exposure limit of 50 ppm over 8 h per working day or 40 h per working week.^8^ Thus, gas sensors that are capable of detecting low‐concentration hazardous gases, inorganic or organic vapors, are highly demanded in the fields of safety, comfort, health, environment protection, and energy.^9^, ^10^ Traditional chemiresistor gas sensors based on semiconducting oxides have attracted intensive attention in practical application due to their low cost and small size.^9^ However, the requirement of external heaters to maintain high operation temperature (200–600 °C) not only imposes high energy consumption, but also brings thermal safety problems.^5^, ^9^ Among various gas sensing materials, graphene (Gr) has become a promising candidate recently due to its atom‐thick 2D structure, high surface area, excellent electronic conductivity, low electrical noise, and high sensitivity to electrical perturbations from gas molecules.^11^ The adsorption of trace amounts of gas molecules on Gr surface causes significant charge transfer between Gr and gas molecules, resulting in a noticeable conductance change of Gr.^5^, ^11^ Since the pioneering work reported the capability of Gr to detect a single NO_2_ molecule,^12^ Gr materials fabricated via various strategies, such as mechanical exfoliation,^12^, ^13^, ^14^ chemical vapor deposition,^1^, ^9^, ^15^, ^16^ epitaxial growth,^17^ and chemically^18^, ^19^, ^20^, ^21^ or thermally^22^ reduced graphene oxide (RGO) have been exploited for gas sensing. Among them, RGO has attracted widespread attention for this purpose due to the low cost and high yield in production, and the convenience of modifying it with functional groups or doping atoms to tailor its gas sensing properties.^19^, ^23^ However, the practical application of unmodified RGO sensor is hindered by low sensitivity, slow response, and poor recovery at room temperature.^19^, ^24^

Chemical modification of Gr/RGO is an effective strategy to boost gas detection, including increased sensitivity, accelerated response, recovery, and lowered limit of detection (LOD).^11^, ^19^, ^25^ For instance, a theoretical study (first‐principle calculations) shows that S‐doped Gr is able to chemically bind to NO_2_ molecules strongly because S‐doped Gr shows much higher adsorption energy with NO_2_ molecules than undoped Gr.^26^ This may lead to considerably boosted sensitivity of S‐doped Gr/RGO to NO_2_.^26^ Although a recent experimental study demonstrates that sulfonated RGO (S‐RGO) shows a 16 times higher response to NO_2_ compared with its undoped RGO counterpart,^19^ the reported S‐RGO based gas sensors either suffer from sluggish response, slow recovery, or complicated processes to prepare the sensing materials,^19^, ^21^, ^27^ which limit its practical application. For example, it demands multiple steps and complicated procedures to synthesize S‐RGO and further modify it with metal/metal oxide nanoparticles.^19^, ^21^, ^27^ In addition to chemical modification of sensing materials, the performance of chemical sensors can also be enhanced through newly designed material structures. For example, recently 3D Gr/RGO porous structures have been utilized to significantly improve the gas sensing performance compared with the 2D counterparts.^1^, ^4^, ^15^, ^28^, ^29^ This is because the unique porous structure coupled with the inherent properties makes 3D RGO exhibit a higher surface area and much more “space” for the transportation or storage of electron/hole and gas, leading to an improved sensitivity.^30^

**Figure 1:**
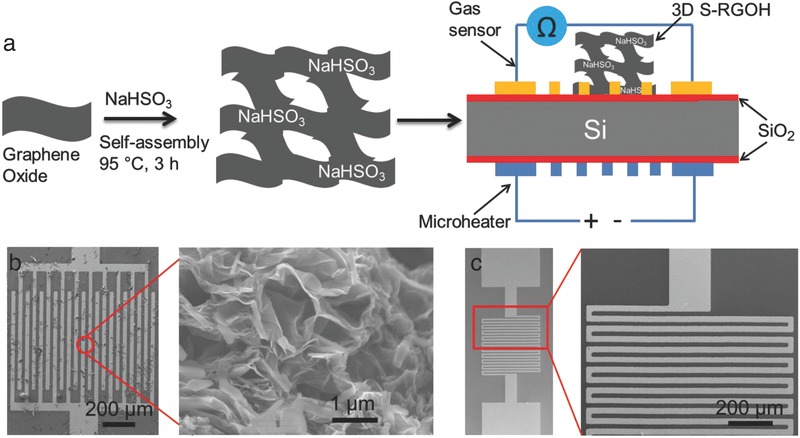
a) Schematic illustrating the synthesis of 3D S‐RGOH in a one‐step self‐assembly process with the aid of NaHSO_3_ and subsequent utilization of the synthesized S‐RGOH for gas detection. A microheater is imbedded at the below side of Si/SiO_2_ substrate to improve the selectivity. b) Scanning electron microscopy (SEM) images revealing that the gaps on the IEs are bridged by the 3D porous S‐RGOH. c) SEM images of a microheater (left) and the magnified serpentine Pt heating lines on the microheater (right).

Although RGO holds great promise in high‐sensitive gas sensing, different gas molecules may adsorb on the same RGO flake surface and change its resistance, leading to a poor selectivity.^31^ The selective detection of a single gas has been reported for Gr/RGO based sensors, but the detection of many different gases selectively by the same sensor is precluded.^11^, ^32^ In many cases, the Gr sensors respond to multiple gases, making it challenging to distinguish them. For example, previously reported S‐RGO‐based gas sensors focus on NO_2_ sensing,^19^, ^21^, ^27^ leaving unexplored the detection of many other important gases such as NH_3_ and volatile organic compounds (VOCs) by S‐RGO sensors. Herein, we report a 3D sulfonated RGO hydrogel (S‐RGOH)‐based gas sensor with boosted performance by combining chemical functionalization and 3D structural modification of RGO with sulfonated groups (**Figure** **1**a). The 3D S‐RGOH is synthesized in a one‐step hydrothermal reaction at low temperature (<100 °C). The 3D S‐RGOH sensor is deployed to detect a variety of important gases, including NO_2_, NH_3_, and VOCs, with high sensitivity, fast response, and full recovery, while possessing the capability of differentiating these gases by elevating substrate temperature via an imbedded microheater, indicative of good selectivity. Compared with its unmodified RGOH counterparts, the S‐RGOH exhibits 118.6 and 58.9 times higher responses to 2 ppm NO_2_ and 200 ppm NH_3_, respectively. In addition, the 3D S‐RGOH sensor displays much lower LOD, faster response, and accelerated recovery compared with the unmodified RGOH counterpart.

## 2 Results and Discussion

### 2.1 Characterization of 3D S‐RGOH

**Figure 2:**
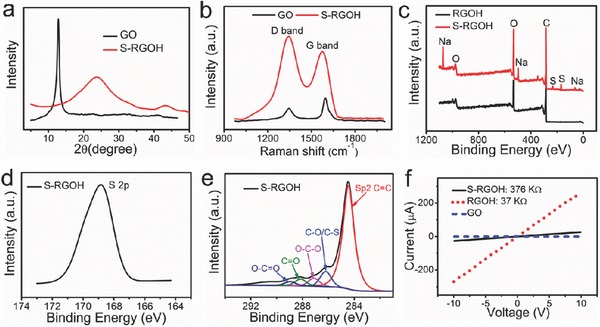
Characterization of 3D S‐RGOH. a) XRD spectra of GO (black) and S‐RGOH (red). b) Raman spectra of GO (black) and S‐RGOH (red). c) XPS survey scan spectra of RGOH (black) and S‐RGOH (red). d,e) High‐resolution S 2p and C 1s XPS spectra of S‐RGOH, respectively. f) Current versus voltage curves of GO (short dashed), RGOH (short dotted), and S‐RGOH (solid).

NaHSO_3_ was exploited to reduce GO and modify RGOH with sulfonated groups simultaneously in a facile, one‐step hydrothermal synthesis process at relatively low temperature (<100 °C) (Figure 1a).^33^ SEM images exhibit 3D porous structures of the synthesized 3D S‐RGOH (Figure 1b). Stacked layers of RGO sheets form the walls of pores with the pore size ranging from tens of nanometers to several micrometers. The X‐ray diffraction (XRD) patterns in **Figure** **2**a confirm the efficient deoxygenation of GO and the formation of S‐RGOH after NaHSO_3_ reduction. The interlayer spacing of S‐RGOH is calculated to be 3.76 Å, which is a little larger than that of graphite (3.35 Å), but much smaller than that of GO (6.95 Å), demonstrating the existence of π–π stacking between RGO sheets in S‐RGOH.^34^ The broad peak at 23.7° on the XRD patterns of S‐RGOH implies that RGO sheets are ordered poorly along their stacking direction and the formation of porous S‐RGOH nanostructures, which is consistent with the SEM characterization in Figure 1b.^34^ Besides, a small peak appears at 43.2°, which is a fingerprint peak of graphite, demonstrating the reformation of graphitic microcrystals on the S‐RGOH plane.^33^ The formation of S‐RGOH is further consolidated by Raman spectra analysis (Figure 2b). A D‐band (1343 cm^−1^) and a G‐band (1593 cm^−1^) appear on the Raman spectra of GO and S‐RGOH. The D‐band implies the presence of the structural defects or attachments of functional groups on the carbon basal plane, which agrees well with previous study.^19^, ^33^ The G‐band is associated with the first‐order scattering of the E2g mode.^19^ The increased ratio of *I*_D_/*I*_G_ from GO to S‐RGOH suggests the formation of new sp^2^ clusters after NaHSO_3_ reduction.^19^ After removal of unreacted NaHSO_3_ in the aqueous S‐RGOH solution by centrifugation and washing several times with deionized (DI) water, the elemental analysis of X‐ray photoelectron spectroscopy (XPS) was carried out (Figure 2c–e). It indicates that S‐RGOH is composed of C, O, S, and Na and that the unmodified RGOH consists only of C and O, demonstrating that NaHSO_3_ has been successfully functionalized on the S‐RGOH surface. Quantitative XPS analysis of the contents of the three elements of C, O, and S reveals that the S‐RGOH has a relatively high content of C atoms (72.7%), a small proportion of O atoms (23.7%), and some S atoms (3.6%) (Table S1, Supporting Information). The ratios of C/O and C/S were calculated to be 3.1 and 20.2, respectively, reflecting that twenty C atoms were modified with one HSO_3_^−^ group on average.

The synthesized 3D S‐RGOH was uniformly dispersed in water under ultrasonication. After depositing the aqueous dispersion of S‐RGOH on Au interdigital electrodes (IEs) by drop‐casting, the S‐RGOH bridged the gaps on the Au IEs. The Au IEs and microheater were fabricated by micromachining technologies. The current versus voltage (*I*–*V*) curve of the S‐RGOH in Figure 2f demonstrates that the synthesized S‐RGOH is conductive. In contrast, the precursor GO is nearly nonconductive. Note that the device displays a linear ohmic behavior, indicating that the low‐resistance electrical contact is negligible in the gas sensing process.^35^ The absence of Schottky barriers between the Au IEs and S‐RGOH ensures the good evaluation of the intrinsic gas sensing property of the S‐RGOH.^35^ The slopes on the linear *I*–*V* lines demonstrate that the resistance of S‐RGOH (376 kΩ) is higher than that of unmodified RGOH (37 kΩ) at room temperature, which can be attributed to the increased defect sites introduced by NaHSO_3_ molecules.^19^

### 2.2 Gas Sensing Performance

**Figure 3:**
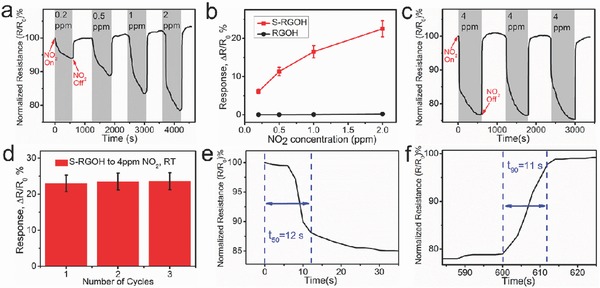
Detection of NO_2_. a) Dynamic response of the 3D S‐RGOH sensor versus time upon exposure to 0.2–2 ppm NO_2_. b) Plots of the quantitative responses of the S‐RGOH and RGOH sensors versus NO_2_ concentration. c,d) Dynamic and quantitative responses of the S‐RGOH sensor upon exposure to 4 ppm NO_2_ repeatedly in three cycles. e,f) Analyses of the response time *t*_50_ and recovery time *t*_90_ in 4 ppm NO_2_ detection in (c), respectively. Gray and white vertical strips denote on and off states of test gas, respectively.

The normalized resistance is defined as *R*/*R*_0_ (%), in which *R* and *R*_0_ are the resistances after and before exposure of the sensor to a test gas, respectively. Furthermore, the response is defined as: (*R*_0_ − *R*)/*R*_0_ (%) = Δ*R*/*R*_0_ (%). It is clear that the normalized resistance of the S‐RGOH sensor decreases rapidly and significantly upon exposure to NO_2_ (**Figure** **3**a). Furthermore, the response increases monotonically from 6.1% at 200 ppb NO_2_ to 22.5% at 2 ppm NO_2_ (Figure 3b). It is worth noticing that the response of the 3D S‐RGOH sensor to 2 ppm NO_2_ is 118.6 times larger than that of the unmodified RGOH counterpart. To evaluate the repeatability of this sensor, the S‐RGOH based sensor was exposed to 4 ppm NO_2_ for three successive cycles (Figure 3c,d). As such, a nearly constant response with a small variation of 2.54% was observed, indicative of good repeatability and stability. To investigate the response and recovery speed, the response time *t*_50_ is defined as the time to achieve 50% of its steady resistance in the response process, and the recovery time *t*_90_ is defined as the time to reach 90% of its original resistance in the signal recovery process after a sensing event.^9^ As such, the *t*_50_ and *t*_90_ of this sensor are calculated to be only 12 and 11 s, respectively, indicating fast response and recovery. Moreover, the sensor displays 100% signal recovery at room temperature, bypassing the demand of UV illumination or thermal treatment to facilitate the signal recovery. The good reversibility displayed by this 3D S‐RGOH‐based NO_2_ sensor is different from those of RGO and carbon nanotube based NO_2_ sensors, which usually require UV light or elevated temperature to improve the recovery.^19^ Analysis of the response curve of the S‐RGOH sensor divides it into two stages: rapid and slow response stages, respectively (Figure S1, Supporting Information).^14^ The response in the rapid response stage produced the *t*_50_ as short as 12 s. The fast response was attributed to molecular adsorption on low‐energy binding sites, such as sp^2^‐bonded carbon. In the slow response stage, structural defects or functional sulfonated groups on S‐RGOH surface reacted slowly with NO_2_ molecules, leading to prolonged response time.^1^ The stabilized/saturated response to 0.2 ppm NO_2_ can be obtained by prolonging the NO_2_ exposure time to 900 s (Figure S2, Supporting Information).

Notice that the S‐RGOH sensor also displayed a linear response versus NO_2_ concentration relationship in the detection of NO_2_ with higher concentrations from 6 to 13 ppm (Figure S3, Supporting Information). The LOD can be calculated from the linearly fitted response versus NO_2_ concentration line (Figure S4, Supporting Information). The LOD of a sensor can be obtained when the noise level is lower than one third of the signal level.^1^, ^20^, ^36^ At the LOD concentration, an analytical signal needs to be differentiated clearly from noise. From the slope (sensitivity) of the linearly fitted response versus concentration relationship and the root‐mean‐square deviation (noise level) at the baseline of response curves before NO_2_ exposure, the sensitivity, noise level, and theoretical LOD can be extrapolated (Figure S4 and Tables S2 and S3, Supporting Information). As such, the sensitivity of this 3D S‐RGOH sensor was calculated to be 8.69 ppm^−1^, which was 65.3 times larger than that of RGOH sensor (0.133 ppm^−1^).^4^ The sensitivity/response displayed by this S‐RGOH sensor is also higher than those of many reported NO_2_ sensors based on Gr/RGO materials (sensitivity: 0.001–0.443 ppm^−1^) (Table S4, Supporting Information).^19^, ^29^ It is noteworthy that the theoretical LOD of the S‐RGOH sensor was calculated to be as low as 4.1 ppb, which was much lower than that of the unmodified RGOH counterpart (178 ppb).^4^ According to the American Conference of Governmental Industrial Hygienists, a threshold exposure limit of 200 ppb NO_2_ is recommended.^5^ Apparently, the theoretical LOD of our sensor is far below the threshold exposure limit. Furthermore, as mentioned above, this sensor exhibits a clear response of 6.1% to 200 ppb NO_2_, which is the lowest NO_2_ concentration provided by our current experimental setup. The ability to detect NO_2_ with low concentration indicates the potential practical applicability of this gas sensor.

The improved response of the S‐RGOH sensor compared with its unmodified counterpart is ascribed to the strong interaction between sulfonated functional groups and NO_2_ molecules. The HSO_3_^−^ is an electron‐withdrawing group and has many lone pairs of electrons.^19^, ^27^ Gas molecules such as electron‐withdrawing NO_2_ tend to adsorb on electron‐rich sites of sulfonated groups.^19^, ^27^ Upon NO_2_ adsorption, the electron transfer from p‐type S‐RGOH to NO_2_ leads to a higher concentration of holes in S‐RGOH, reducing the resistance of S‐RGOH. Furthermore, Dai et al. predict that S‐doped Gr is able to chemically bind NO_2_ strongly based on first‐principle calculations.^26^ Due to the weak interaction between the gas molecules and the lone‐pair electrons in HSO_3_^−^ groups, gas molecules can be desorbed from the S‐RGOH surface facilely in the air purge process. In addition to functional HSO_3_^−^ groups, the sensitivity is also enhanced by the pore‐filling of NO_2_ in the 3D porous structures of S‐RGOH, which provides increased adsorption site density.^37^ The NO_2_ sensing performance of the sensor in high relative humidity (RH) condition is also studied (Figure S5, Supporting Information). Due to the interference of water molecules, the response to 10 ppm NO_2_ deteriorated 30% from 0% to 70% RH. This is because the electron‐withdrawing NO_2_ and electron‐donating H_2_O molecules make the resistance of p‐type S‐RGOH decrease and increase, respectively. However, the prehumidification treatment of S‐RGOH can be employed to suppress the interference of humidity on NO_2_ detection.^8^ After prehumidification treatment of the sensor in 70% RH for 20 min, 10 ppm NO_2_ gas was introduced in the chamber at 70% RH. It was found that the response only reduced 7.1% in this case as compared with that at 0% RH. The prehumidification makes the response of the sensor to 70% RH reach a saturated state before NO_2_ exposure and therefore the sensor becomes less vulnerable to high RH.^8^ In practical gas sensing application, the prehumidification can happen naturally on the sensor at high RH. It demonstrates that the S‐RGOH sensor has good ability to endure high RH.

**Figure 4:**
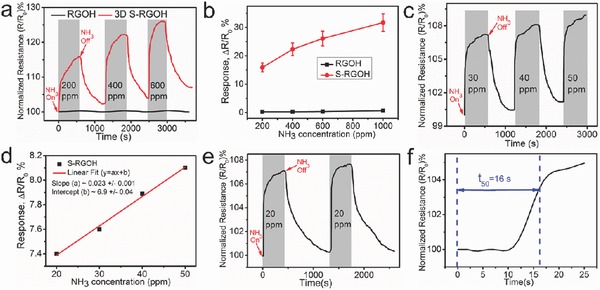
NH_3_ sensing. a) Dynamic responses of the 3D S‐RGOH and RGOH sensors to 200–600 ppm NH_3_. b) Plots of response versus NH_3_ concentration for the S‐RGOH and RGOH sensors. c) Dynamic response of the S‐RGOH sensor to NH_3_ with low concentration from 30 to 50 ppm. d) Plots of experimentally obtained and linear fitted response versus NH_3_ concentration. e) Response of the S‐RGOH sensor to 20 ppm NH_3_ in two successive cycles. f) Analysis of the response time *t*_50_ of the S‐RGOH sensor to 20 ppm NH_3_.

In addition to NO_2_, this 3D S‐RGOH sensor is also highly responsive to NH_3_. The sensor displays fast and substantial increase in its resistance upon exposure to 20–1000 ppm NH_3_ (**Figure** **4**). The response of the S‐RGOH sensor to 200 ppm NH_3_ is 58.9 times larger than that of the unmodified RGOH counterpart. Notice that the S‐RGOH sensor shows a nearly linear relationship between response and NH_3_ concentration (Figure 4a–d), from which the theoretical LOD of NH_3_ detection can be deduced to be as low as 1.48 ppm (Figure S6 and Tables S5 and S6, Supporting Information). Although the lowest NH_3_ concentration limited by our current gas sensing setup is 20 ppm (Figure 4e), NH_3_ with lower concentration may also be detected by our sensor in future work based on the theoretically calculated LOD. The ability to detect NH_3_ lower than 25 ppm is significant for practical application.^1^, ^38^ Apparently, the S‐RGOH sensor displays a clear response of 7.1% to 20 ppm NH_3_, lower than the criteria of 25 ppm. Similar to the response to NO_2_, the response curves of this sensor to NH_3_ can be divided into two parts, an initial fast response phase followed by slow response period, which are attributed to NH_3_ adsorption on low‐energy and high‐energy binding sites, respectively (Figure S7, Supporting Information). Thus, the response time *t*_50_ of this sensor to 20 ppm NH_3_ is only 16 s, revealing the rapid response of the sensor (Figure 4f). In comparison with other Gr/RGO based NH_3_ sensors, this S‐RGOH sensor displays faster response while preserving comparable sensitivity (Table S7, Supporting Information). When the sensor was exposed to 200 ppm NH_3_ in three successive cycles to study the repeatability, a constant response of 13% with a small variation of 3.8% was observed (Figure S8, Supporting Information). The sensor exhibited full recovery in each cycle, indicative of good reversibility. To investigate the effect of NH_3_ exposure time on the response, we found that the response increased monotonically with prolong NH_3_ exposure time until the sensor reached the stabilized/saturated response to 200 ppm NH_3_ at 800 s (Figure S9, Supporting Information).

Different from the electron‐withdrawing nature of oxidizing NO_2_ gas, NH_3_ is an electron‐donating reducing gas.^1^ Upon the adsorption of NH_3_ molecules on S‐RGOH surface, the electron transfer from NH_3_ to S‐RGOH reduced the carrier (hole) concentration in S‐RGOH. The hole‐depletion in p‐type S‐RGOH leads to an increased resistance. The boosted NH_3_ sensing response of S‐RGOH compared with unmodified RGOH is attributed to the chemical reaction between sulfonic groups and NH_3_, forming ammonium salts.^8^, ^37^, ^39^ After exhaustion of reactive surface sites, weak interactions, including hydrogen bonding, dipole/dipole, and dispersive interactions, dominate the adsorption.^37^ Hydrogen bonding can form between HSO_3_^−^ groups and NH_3_. NH_3_ molecules can also fill in the pores of 3D S‐RGOH, increasing the interaction area. Furthermore, the hopping of charge carriers across the micro/nanoscale pores offers alternative paths for charge transport, leading to a greater signal level.^37^ Similar to NH_3_, the electron‐donating water molecules also cause the increase of resistance of S‐RGOH. Consequently, the response of the S‐RGOH sensor to 30 ppm NH_3_ at 70% RH was enhanced 118% due to the overlapped response from humidity (Figure S10, Supporting Information). Nevertheless, the response of the sensor to 30 ppm NH_3_ only increased 6% from 0% to 70% RH after prehumidification treatment.

**Figure 5:**
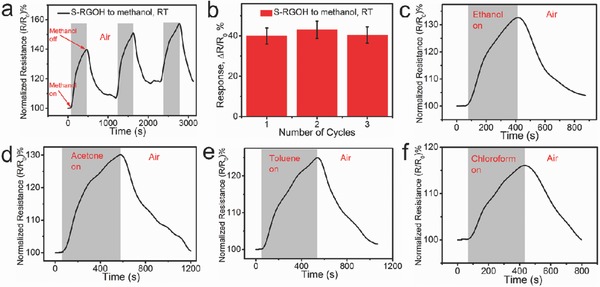
Responses of the S‐RGOH sensor to various saturated organic vapors, including a) 5.5 ppb methanol, c) 3.8 ppb ethanol, d) 3 ppb acetone, e) 2.1 ppb toluene, and f) 2.8 ppb chloroform. b) Quantitative response of this sensor to saturated methanol vapor in three successive cycles. The calculation of the concentration of these VOCs is shown in supporting text and Table S8 (Supporting Information).

Alcohol sensors with high sensitivity and good stability are always in great demand in chemical, biomedical, and food industries, especially in breath and wine‐quality analysis.^10^, ^40^ In addition to NO_2_ and NH_3_, the S‐RGOH sensor also exhibits high sensitivity to a variety of VOCs. For example, the responses of this sensor to saturated vapors of methanol, ethanol, acetone, toluene, and chloroform reached as high as 40%, 33%, 30%, 25%, and 16.2%, respectively (**Figure** **5**). A constant response of 40% with a variation of 4.1% was observed when the sensor was exposed to a saturated methanol vapor for three successive cycles, indicating good repeatability and stability (Figure 5a,b). The response displayed by this 3D S‐RGOH sensor to organic vapors is comparable to that of the previous reported RGO/Gr‐based sensors.^14^, ^20^, ^27^, ^41^ Compared with its unmodified counterpart, the S‐RGOH sensor exhibits more than two orders of magnitude higher responses to these organic vapors,^27^ demonstrating the effectiveness of chemical functionalization in improving its sensitivity. The reduction in the resistance when the S‐RGOH sensor was exposed to an electron‐withdrawing gas such as NO_2_ was opposite to the increase in resistance when it was exposed to electron‐donating saturated VOCs, such as CH_3_OH, C_2_H_5_OH, and CHCl_3_, which agrees well with previous studies.^20^ Although our current experimental facilities are inadequate to adjust the concentration of organic vapors, the good response of this sensor to VOCs is extremely encouraging. In our future work, we will attempt to address the LOD of VOC sensing and engineer the content of functional groups to optimize the sensitivity.

### 2.3 Improved Selectivity by Microheater

**Figure 6:**
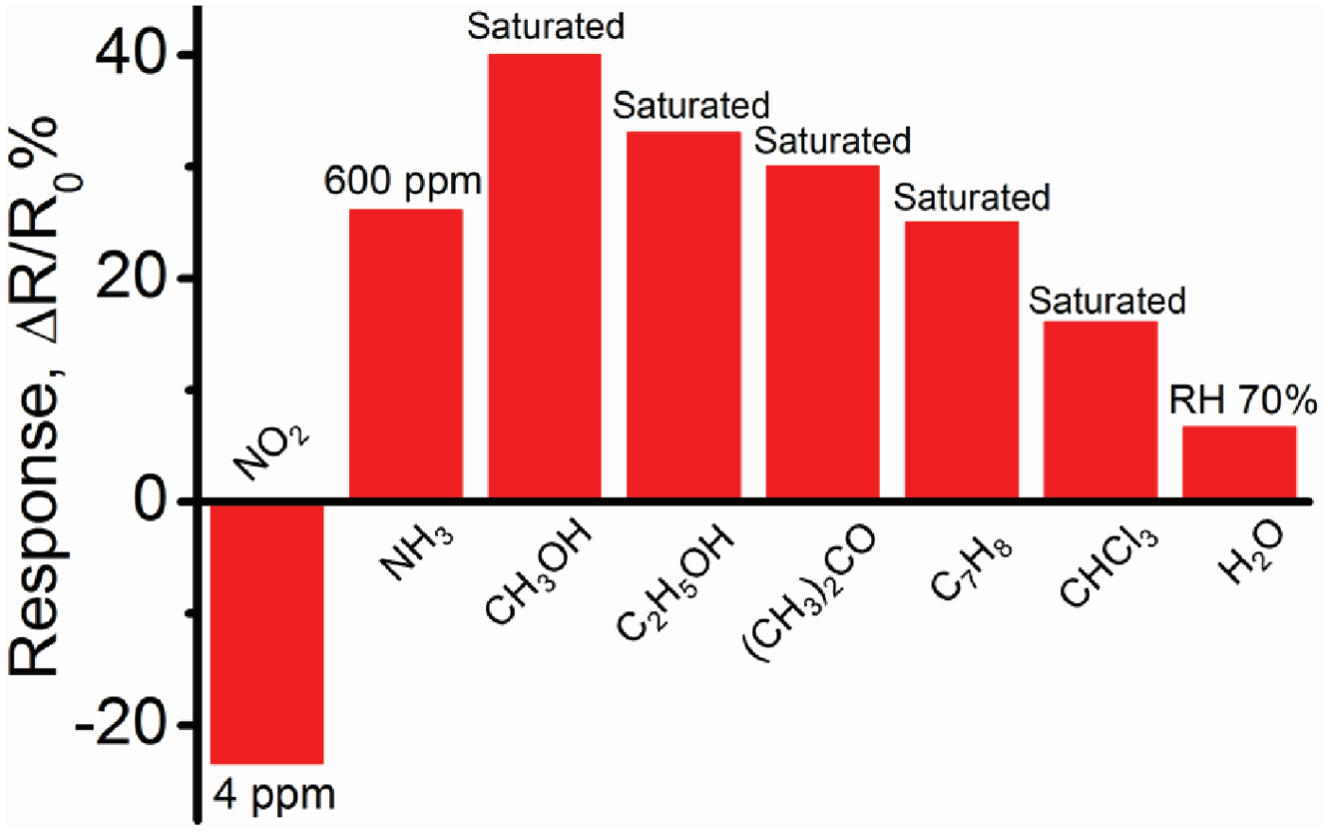
Responses of the S‐RGOH sensor to various gaseous chemicals, including NO_2_ (4 ppm), NH_3_ (600 ppm), saturated methanol, ethanol, acetone, toluene, chloroform vapors, and 70% RH.

**Figure 7:**
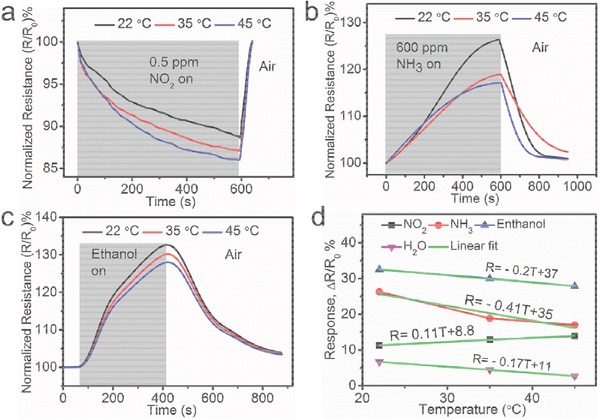
Discrimination of various gases by raising substrate temperature via an imbedded microheater. a–c) Responses of this S‐RGOH sensor to NO_2_ (0.5 ppm), NH_3_ (600 ppm), and ethanol (3.8 ppb) at 22, 35, and 45 °C, respectively. d) Plots of experimental obtained and linearly fitted responses of this sensor to NO_2_ (0.5 ppm), NH_3_ (600 ppm), ethanol (3.8 ppb), and 70% RH versus temperature.

Previously reported Gr/RGO based gas sensors suffer from the inability to detect and distinguish different gases, leading to a poor selectivity.^11^, ^31^ Here, although the S‐RGOH sensor exhibits high sensitivity to various gaseous chemicals (**Figure** **6**), we can utilize the characteristic patterns on the response versus temperature curves to discriminate these gases successfully for the first time (**Figure** **7**). Specifically, we found that the response of the sensor with change in temperature varied for different gases. For NO_2_ sensing, the response increased slightly with the elevated temperature from 22 to 45 °C, from which the response–temperature relationship could be linearly fitted as *R* = 0.11*T* + 8.8 (Figure 7a,d). However, the response decreased when the temperature was further raised to 65 °C by an imbedded microheater (Figure S11, Supporting Information). In contrast, the responses to NH_3_, ethanol, and water declined with temperature (Figure 7b–d and Figure S12 (Supporting Information)). The response–temperature curves for NH_3_, ethanol, and 70% RH detection were linearly fitted as *R* = −0.41*T* + 35, *R* = −0.2*T* + 37, and *R* = −0.17*T* + 11, respectively (Figure 7d). Apparently, the slopes and intercepts on the linearly fitted response lines for these four different chemicals are totally different (**Table** **1**). Note that the distinctive slopes and intercepts can be exploited to conveniently differentiate the four different gases. Previously, we have demonstrated that an integrated microheater can be employed to differentiate NO_2_ and NH_3_ since the elevated temperature does and does not suppress the response of the RGOH sensor to NH_3_ and NO_2_, respectively.^4^ However, this method is only applicable to two different gases. Here, we found that many different gases had their unique features such as slope and intercept on the linear fitted response–temperature lines, which could be employed to distinguish these gases. Although we have only chosen four representative gases to demonstrate the selectivity, in principle, we can extend this method to sort out a variety of different gases since each gas has its unique response–temperature patterns, leading to an enhanced selectivity for each detectable gas. This enables the S‐RGOH sensor to detect many different gases with simultaneously high sensitivity and good selectivity.

**Table 1:** Linear fitted response–temperature relationships for four different gaseous chemicals

	Linear fitted equation	Slope	Intercept
NO_2_	*R* = 0.11*T* + 8.8	0.11	8.8
NH_3_	*R* = −0.41*T* + 35	−0.41	35
Ethanol	*R* = −0.2*T* + 37	−0.2	37
H_2_O	*R* = −0.17*T* + 11	−0.17	11

The temperature of the sensor was elevated rapidly by a miniaturized microheater imbedded on the substrate of the sensor (Figure S13, Supporting Information).^4^ The substrate temperatures were 22, 35, 45, and 65 °C when the direct current (DC) voltages of 0, 10, 15, and 20 V were applied to the microheater, respectively. The resistance of S‐RGOH stabilized within 60 s after a DC voltage of 15 V was applied (Figure S14a, Supporting Information). Furthermore, the resistance of S‐RGOH could recover to the original value within 50 s after the DC voltage was turned off, demonstrating the fast and convenient modulation of sensor temperature by the microheater. With such a rapid manipulation of sensor temperature, the sensor could be potentially deployed to screen and recognize a series of different detectable gases in future work. The resistance of S‐RGOH declined rapidly with elevated temperature (Figure S14b, Supporting Information), indicative of negative temperature coefficient and semiconducting behavior of S‐RGOH (dominated by thermally activated charge carriers).^42^ Note that the power consumptions of both the microheater and the chemiresistor are small, demonstrating the energy‐saving advantage of this device (Figure S15, Supporting Information).

The varied response–temperature relationship for different gases should be attributed to different adsorption energy (*E*_ad_) between S‐RGOH and gas molecules.^4^ For example, a first‐principle study indicates that S‐doped Gr shows much higher adsorption energy with NO_2_ molecules (−0.831 eV) than NH_3_ molecules (−0.003 eV) and H_2_O molecules (−0.019 eV).^26^ Furthermore, the equilibrium distance of S‐doped Gr—NO_2_ distance (1.47 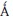) is much smaller than that of S‐doped Gr—NH_3_ (4.03 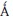) and that of S‐doped Gr—H_2_O (3.88 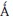).^26^ These demonstrate that S‐doped Gr displays stronger interaction with NO_2_ compared with NH_3_ and H_2_O molecules. Therefore, the elevated temperature within proper range weakens the response of this Gr sensor to NH_3_ and H_2_O molecules, but not to NO_2_. There is a competition between adsorption and desorption of NO_2_ molecules on S‐RGOH surface during the NO_2_ sensing process.^2^ At low temperature (22–45 °C), the increased adsorption of NO_2_ dominates, while desorption is negligible due to the strong bonding between S‐doped Gr and NO_2_.^2^ Furthermore, the elevated temperature activates the charge carriers.^43^ Therefore, the response of S‐RGOH to NO_2_ increased slightly from 22 to 45 °C. However, some reactions between electron‐withdrawing O_2_ and electron‐rich HSO_3_^−^ groups may happen at further elevated temperature to 65 °C, leading to a reduced response. It demonstrates that an appropriate temperature is important to optimize the sensitivity of S‐RGOH sensor. To the best of knowledge, this is the first time that the locally elevated temperature is employed to enhance the selectivity of detecting various different gases. This method may shed light on the improvement of the selectivity of various chemical sensors by programing the temperature of sensing materials in future work.

## 3 Conclusions

In summary, we have developed a simple chemiresistor‐type gas sensor based on 3D sulfonated RGOH with high sensitivity, good selectivity, fast response, and good reversibility toward several gases. Compared with the unmodified RGOH counterpart, the 3D S‐RGOH sensor exhibits more than two orders of magnitude higher response to NO_2_ and VOCs and a 58.9 times higher response to NH_3_. The low theoretical LODs of 4.1 ppb and 1.48 ppm are obtained for NO_2_ and NH_3_ detection, respectively. Notice that the sensor can achieve fast response and complete recovery at room temperature, bypassing the assistance of UV illumination and thermal treatment. The stability and reversibility of the sensor are evidenced by repeated sensing with nearly constant response. In comparison with previously reported sulfonated RGO sensor for single gas detection, the S‐RGOH sensor developed here can detect a variety of different gases with improved selectivity. For example, different gaseous chemicals can be detected and distinguished simultaneously by recognizing the characteristic patterns on the response–temperature curves. The facile synthesis and functionalization of 3D RGOH in one step make it attractive for cost‐effective gas sensing application. This work not only indicates the improvement of sensitivity of gas sensing by combining 3D structure design and chemical functionalization of RGO, but also sheds light on boosting the selectivity of RGO sensors by temperature modulation of RGO using microheater.

## 4 Experimental Section

*Synthesis of GO, S‐RGOH, RGOH, and Fabrication of Gas Sensors*: A modified Hummers' method was deployed to synthesize GO from graphite powder with the details described in the literature.^4^, ^23^ For one‐step synthesis of S‐RGOH, 42 mg reducing agent NaHSO_3_ was added in 10 mL 2 mg mL^−1^ aqueous suspensions of GO. The mixed suspension was heated at 95 °C for 3 h without stirring. The synthesis of unmodified RGOH was described in the previous work.^4^, ^44^ After cooling, the synthesized S‐RGOH was centrifuged and washed several times with DI water. Subsequently, the solid S‐RGOH was collected and redispersed in water to prepare a 2 mg mL^−1^ aqueous dispersion of S‐RGOH. After ultrasonication for 10 min, the obtained aqueous dispersion of S‐RGOH was dispersed uniformly in water.

*Microheater and Sensor Fabrication*: The Au IEs and microheater were fabricated on the above and below sides of the Si/SiO_2_ substrate, respectively, by micromachining technologies.^4^ The 300 µm thick Si wafer had 260 nm thick SiO_2_ layers on both sides. To pattern the Pt heating lines of the microheater, a layer of AZ 9260 positive photoresist was spin coated on the Si/SiO_2_ wafer surface, followed by a photolithography, sputtering of 5 nm Cr/300 nm Pt, and photoresist lift‐off process. Subsequently, the Au bonding pads were fabricated by another photolithography, sputtering of 5 nm Cr/300 nm Au, and lift‐off process. To make the Pt heating lines generate the majority of heat, the width of the Au bonding pads (1 mm) was much larger than that of Pt lines (25 µm). The Au IEs were fabricated by another photolithography, sputtering of 5 nm Cr/100 nm Au, and lift‐off process. Both the width and gap of Au strips on the IEs were 20 µm. After drop casting of the aqueous dispersion of S‐RGOH on the Au IEs and drying, the 3D S‐RGOH bridged the gaps on the IEs and thus could be employed for gas sensing.

*Material Characterizations*: FE‐SEM 7600 was utilized to characterize the morphology of 3D S‐RGOH, Au IEs, and microheater. X‐ray powder diffraction spectra of the prepared S‐RGOH were obtained by a Bruker D8 advance X‐ray diffractometer. Raman spectra of samples were acquired using a spectrophotometer (alpha300 R from WITec) with a 514 nm laser. XPS spectra were obtained using a Kratos XSAM 800 spectrometer with a Mg Kα (1253.6 eV) X‐ray source.

*Gas Sensing Test*: A fixed voltage (0.1 V) was applied on the sensor and the resistance change was monitored by a Keithley 2602 SourceMeter. Dry synthetic air was utilized to dilute the test gas to the desired concentration and also clean the gas chamber before and after test gas exposure. The VOCs were delivered by a room‐temperature saturated vapor stream method. The sensing test was performed on the same sample for five times to obtain the average values and the standard deviation of responses or recovery percent. The gas sensing properties were measured at 22 °C in ambient air, if without special notification.

## Supporting information

As a service to our authors and readers, this journal provides supporting information supplied by the authors. Such materials are peer reviewed and may be re‐organized for online delivery, but are not copy‐edited or typeset. Technical support issues arising from supporting information (other than missing files) should be addressed to the authors.

Supplementary
