# Supplementary material for: A 3D Chemically Modified Graphene Hydrogel for Fast, Highly Sensitive, and Selective Gas Sensor
Source: Adv Sci (Weinh). 2016 Dec 20;4(3):1600319. doi: 10.1002/advs.201600319 (PMC5357982; doi:10.1002/advs.201600319)
Supplement: Supplementary file 1 — Supplementary [file ADVS-4-na-s001.pdf]

Copyright WILEY-VCH Verlag GmbH & Co. KGaA, 69469 Weinheim, Germany, 2016.

ADVANCED SCIENCE
Open Access

Supporting Information

for *Adv. Sci.,* DOI: 10.1002/advs.201600319

A 3D Chemically Modified Graphene Hydrogel for Fast, Highly Sensitive, and Selective Gas Sensor

Jin Wu, Kai Tao, Yuanyuan Guo, Zhong Li, Xiaotian Wang, Zhongzhen Luo, Shuanglong Feng, Chunlei Du, Di Chen, Jianmin Miao,* and Leslie K. Norford*

Supporting Information

3D Chemically Modified Graphene Hydrogel for Fast, Highly Sensitive and Selective Gas Sensor

Jin Wu, Kai Tao, Yuanyuan Guo, Zhong Li, Xiaotian Wang, Zhongzhen Luo, Shuanglong Feng, Chunlei Du, Di Chen, Jianmin Miao* and Leslie K. Norford*

Dr. J. Wu, Dr. K. Tao, Dr. Z. Li, Prof. J. M. Miao
School of Mechanical and Aerospace Engineering, Nanyang Technological University, Singapore 639798, Singapore
E-mail: MJMMiao@ntu.edu.sg

Prof. S. L. Feng, Prof. C. L. Du
Micro-nano manufacturing and system integration center, Chongqing Institute of Green and Intelligent Technology, Chinese Academy of Sciences, Chongqing 400714, PR China
Emails: fengshuanglong@cigit.ac.cn

Prof. D. Chen
Key Lab. for Thin Film and Microfabrication Technology of Ministry of Education,
Department of Instrument Science and Engineering, School of Electronic Information and Electrical Engineering, Shanghai Jiao Tong University, 800 Dongchuan Road, Shanghai 200240, P.R. China
Shanghai Engineering Research Center for Intelligent Diagnosis and Treatment Instrument, 800 Dongchuan Road, Shanghai 200240, P. R. China

Prof. L. K. Norford
Center for Environmental Sensing and Modeling (CENSAM), Singapore-MIT Alliance for Research and Technology (SMART) Centre, Singapore 117543, Singapore
Department of Architecture, Massachusetts Institute of Technology, Cambridge, MA 02139, USA

Y.Y. Guo, Dr. X. T. Wang, Dr. Z. Z. Luo
School of Materials Science and Engineering, Nanyang Technological University, 50 Nanyang Avenue, Singapore 639798, Singapore

Table S1. XPS elemental analysis of 3D S-RGOH

Sample	C	O	S	C/O	C/S
S-RGOH	72.7	23.7	3.6	3.1	20.2

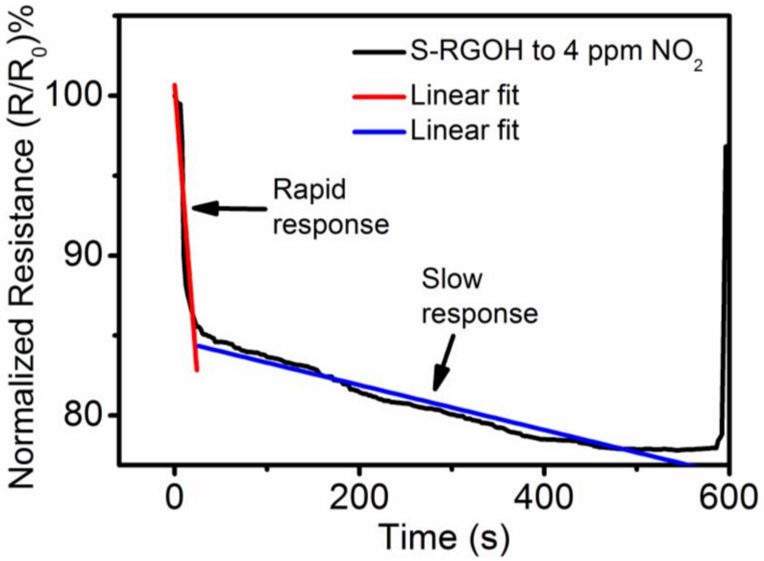

Figure S1. Analysis of the response of the S-RGOH sensor to 4 ppm NO_2 divides it into two stages: rapid response stage (with large slope) and slow response stage (with small slope). The red and blue linear fitted lines correspond to rapid and slow response stages respectively.

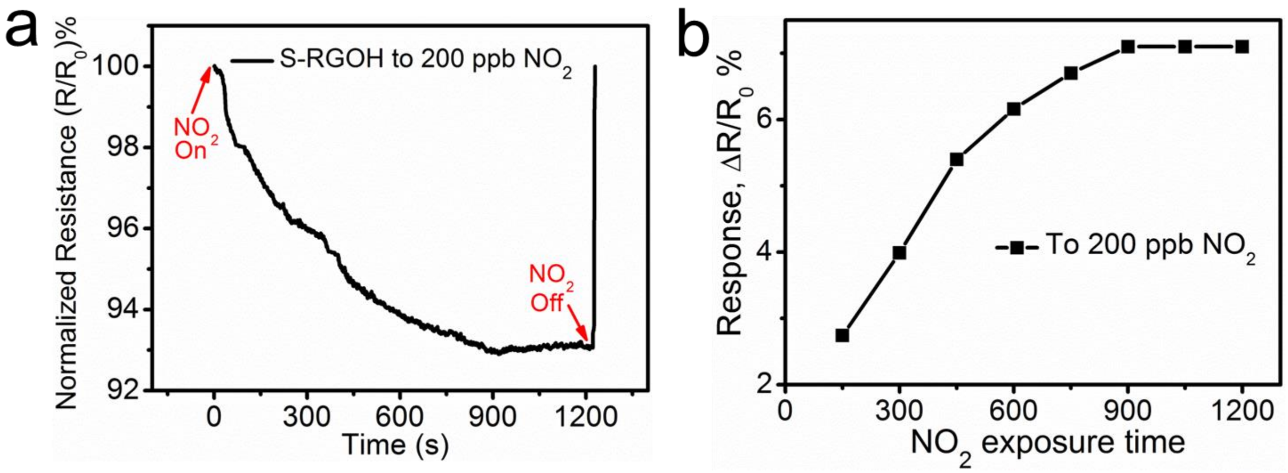

Figure S2. (a) and (b) Dynamic and quantitative responses of the S-RGOH sensor to 200 ppb NO_2 respectively with prolonged NO_2 exposure time to 1210 s. The saturated response of 7.1% was achieved at 900 s.

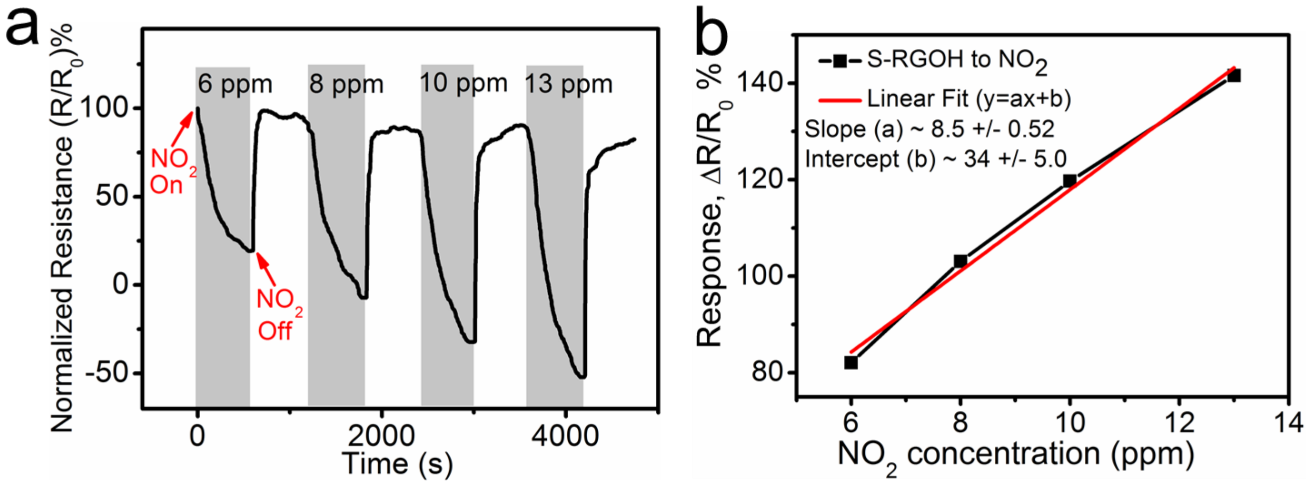

Figure S3. (a) Dynamic sensing response of the S-RGOH sensor to NO_2 with the concentration monotonically increased from 6 to 13 ppm. (b) Plots of experimentally obtained and linearly fitted response of the sensor versus NO_2 concentration.

Calculation of sensitivity, noise level (RMS_{Noise}) and LOD of the 3D S-RGOH based NO_2 sensor.[1, 2]

Step 1: Plot the response versus NO_2 concentration curve, followed by performing the linear fitting of the curve. The slope (sensitivity) and standard error can be obtained from the fitted line (Figure S4a).

Step 2: Implement 5th order polynomial fit for the response ΔR/R% versus time curves at the baseline before NO_2 exposure (Figure S4b).

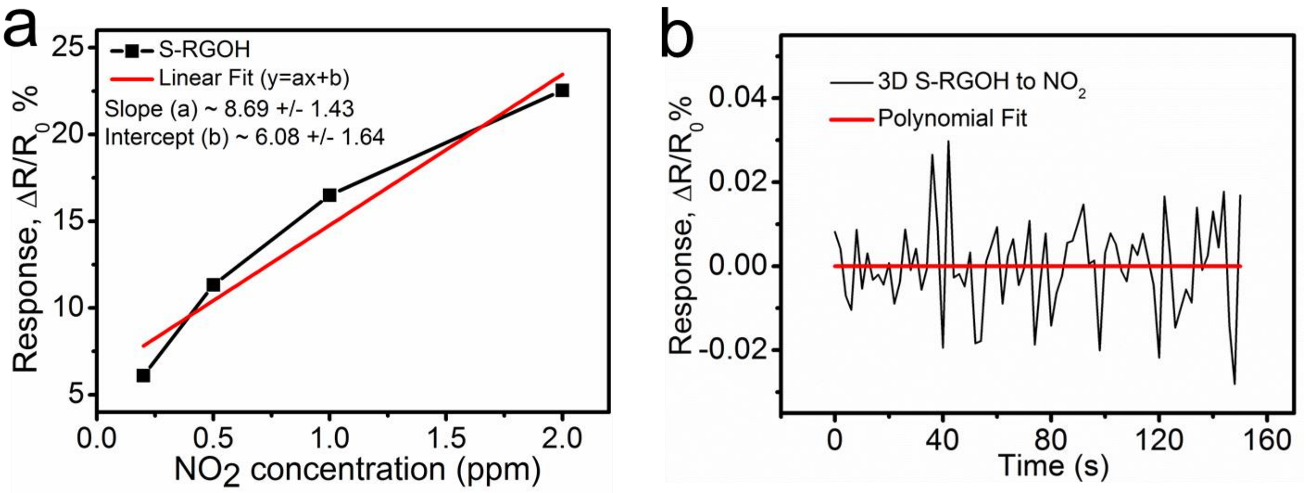

Figure S4. a) The experimentally obtained response (dashed black line) and linear fitted response (red line) as a function of NO_2 concentration. b) Plots of 5th order polynomial fitted response of the 3D S-RGOH sensor as a function of time at the baseline before NO_2 exposure.

Step 3: Take 11 data points at the baseline before NO_2 exposure (Table S2).

Step 4: Calculate regular residual (A_i-A) and statistical parameters of 5th order polynomial fit of response ΔR/R% versus time (sec) curves, where A_i and A are the measured data point and corresponding value calculated from the fitted curve respectively (Table S2).

Step 5: Calculate the root-mean squared deviation (RMS_{Noise}) and LOD using the following Equation S1 and S2:

RMS_{Noise} (ppm^{-1}) = $(V_x^2/(N-1))^{1/2}$, where $V_x^2 = \sum(A_i - A)^2$ (Equation S1)

LOD (ppm) = 3 × RMS_{Noise} / Slope (Equation S2)

Table S2. 5th order polynomial fitting data for the response of 3D S-RGOH sensor before exposure to NO_2

Time (sec)	A_i-A	$(A_i\text{-}A)^2$
0	0.00817	6.67E-05
15	-0.00325	1.06E-05
30	0.00415	1.72E-05
45	-0.00268	7.18E-06
60	0.0093	8.65E-05
75	-0.01866	3.48E-04
90	1.03E-02	1.07E-04
105	0.00523	2.74E-05
120	-2.18E-02	4.75E-04
135	-8.81E-04	7.76E-07
150	1.69E-02	2.84E-04

Table S3. Calculation of sensitivity, noise level (RMS_{noise}) and LOD in NO_2 detection

Material for NO_2 Sensing	Slope (Sensitivity) (ppm^{-1})	Standard Error (ppm^{-1})	V_x^2	RMS_{noise} (%)	LOD (ppm)
3D S-RGOH	8.69	1.43	1.43E-03	0.012	4.13E-03

Table S4. Comparison between different Gr-based NO_2 sensors in terms of sensitivity, response and recovery time

Sensing materials	NO_2 concentration (ppm)	Response	Response time	Recovery time	References
3D S-RGOH	4	$\Delta R/R_a$ (%) = 23.5%	t_{50}=12 s	t_{90}=11 s	Current work
2D RGO (Chemical reduction)	5	$\Delta R/R_a$ (%) = 12%	10 min	10 min	[3]
2D RGO (Heat reduction)	100	$\Delta R/R_a$ (%) = 9.15%	15 min	20 min	[4]
RGO on 3D pillars	5	$\Delta R/R_a$ (%) = 21%	15 min	12 min	[1]
2D Ag-S-RGO	5	$\Delta R/R_a$ (%) = 17%	12 s	20 s	[5]
3D CVD Gr foam	100	$\Delta R/R_a$ (%) = 8%	400 s	400 s	[6]
3D-RGO-SnO_2	50	$\Delta R/R_a$ (%) = 6.5%	250 s	500 s	[7]

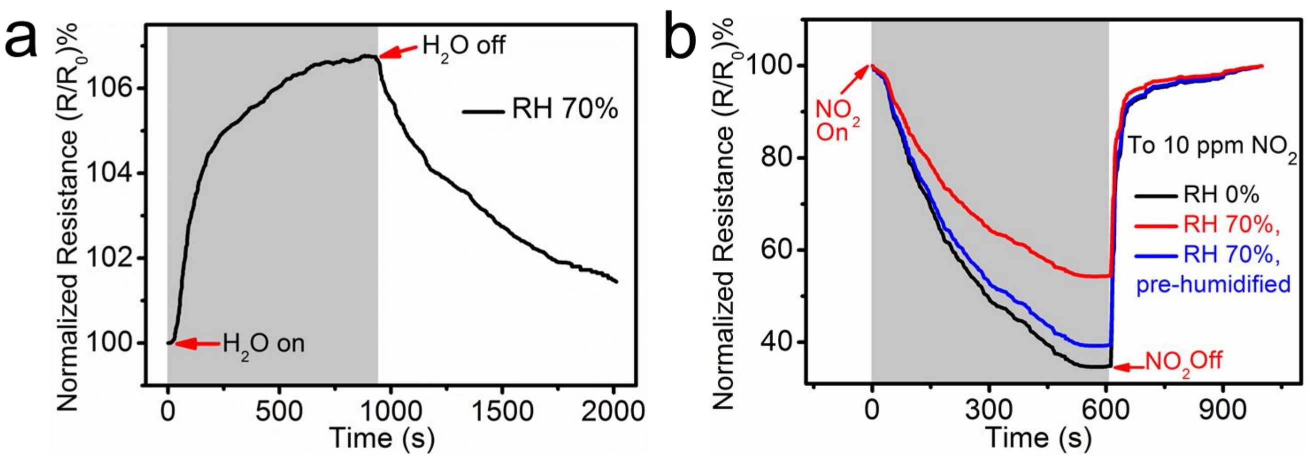

Figure S5. (a) Response of the S-RGOH sensor to 70% relative humidity (RH). (b) Response of the S-RGOH sensor to 10 ppm NO_2 in the conditions of 0% RH (black), 70% RH without pre-humidification (red) and with pre-humidification (blue) treatment, respectively.

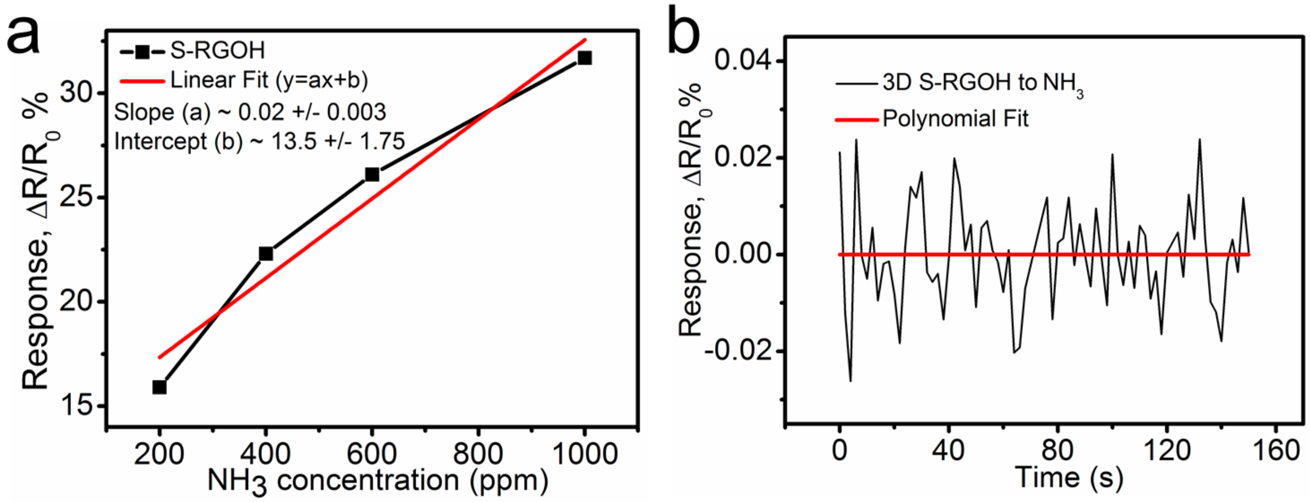

Figure S6. (a) Plots of the experimentally obtained response (dashed black line) and linear fitted response (red line) of the 3D S-RGOH sensor versus NH_3 concentration. (b) Plots of 5th order polynomial fitted response of the sensor versus time at the baseline before NH_3 exposure.

Table S5. 5th order polynomial fitting data for the response of the sensor before exposure to NH_3

Time (sec)	Ai-Ā	(Ai-Ā)^2
0	0.02112498	4.46E-04
15	-0.009503485	9.03E-05
30	-0.003648402	1.33E-05
45	0.014112984	1.99E-04
60	-0.00772434	5.97E-05
75	0.007136502	5.09E-05
90	-1.74E-04	3.04E-08
105	0.002688486	7.23E-06
120	4.58E-04	2.10E-07
135	0.003323978	1.10E-05
150	5.39E-04	2.91E-07

Table S6. Calculation of sensitivity, noise level (RMS_{noise}) and LOD for NH_3 sensing

Material for NH_3 Sensing	Slope (Sensitivity) (ppm^{-1})	Standard Error (ppm^{-1})	V_x^2	RMS_{noise}	LOD (ppm)
3D S-RGOH	0.02	0.003	8.78E-04	9.37E-03	1.48

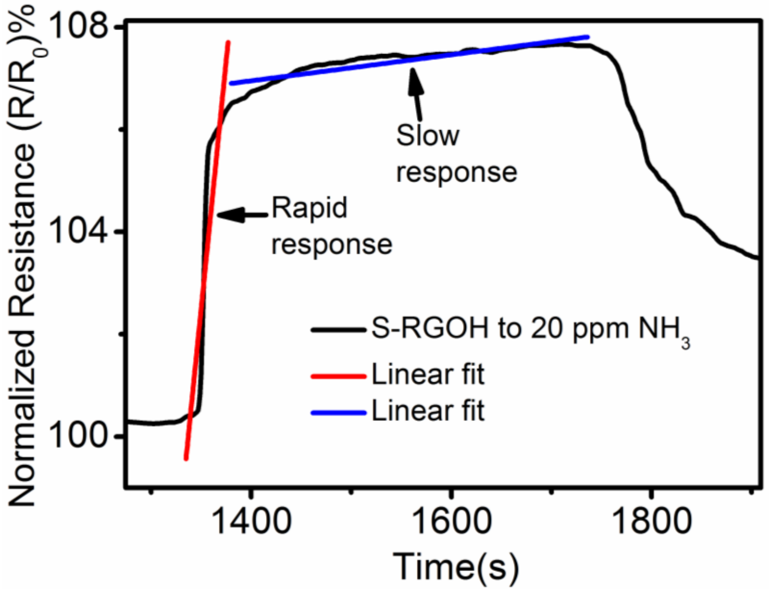

Figure S7. The response of the S-RGOH sensor to 20 ppm NH_3 can be linearly fitted and divided into two parts: the front fast response part (red line) and the latter slow response part (blue line).

Table S7. Comparison of different graphene-based sensors in NH_3 detection

Sensing materials	NH_3 concentration (ppm)	Response	Response time	Recovery time	References
3D S-RGOH	20	$\Delta R/R_a$ (%) = 7%	t_{50}=16 s	10 min	Current work
RGO on 3D pillars	40	$\Delta R/R_a$ (%) = 75%	30 min	90 min	[1]
3D CVD Gr foam	20	$\Delta R/R_a$ (%) = 3%	800 s	800 s	[6]
R-GO on MEMS platform	5	$\Delta R/R_a$ (%) = 2.5%	10 min	10 min	[3]
R-GO/Cu phthalocyanine	50	$\Delta R/R_a$ (%) = 6%	12 min	12 min	[8]

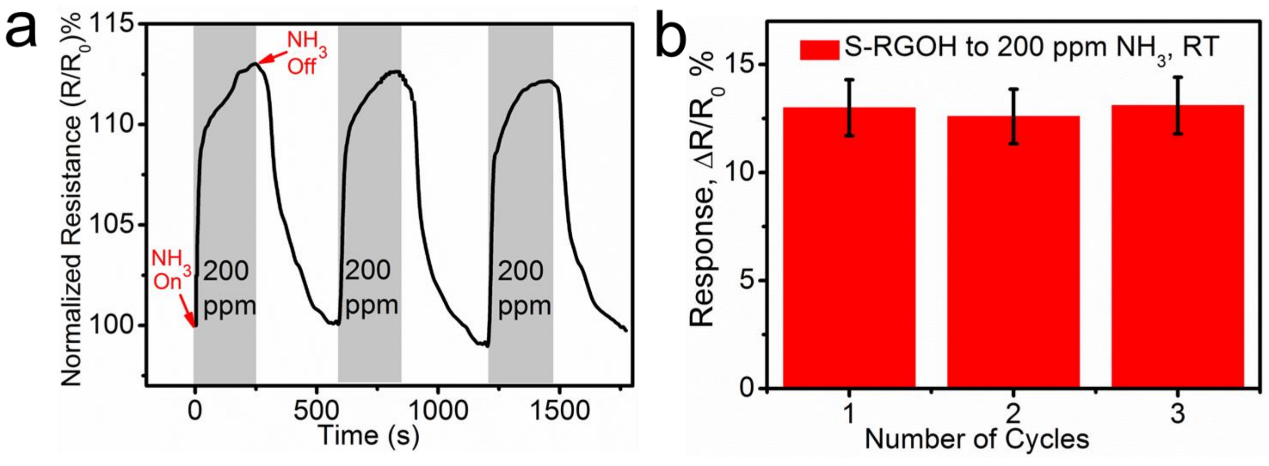

Figure S8. (a) and (b) Dynamic and quantitative responses of this S-RGOH sensor to 200 ppm NH_3 in consecutive three cycles respectively.

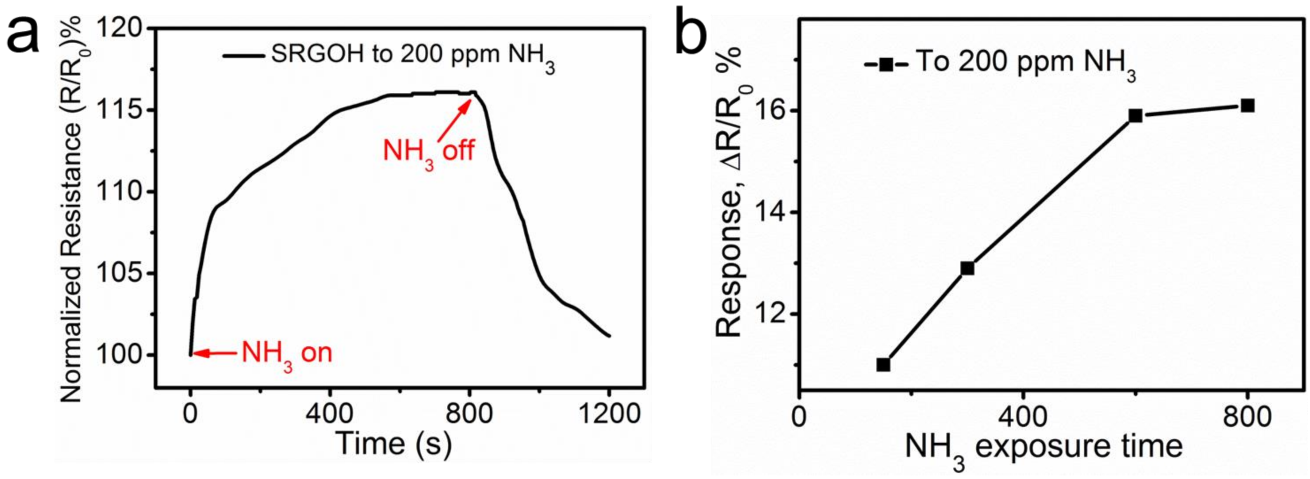

Figure S9. (a) and (b) Dynamic and quantitative sensing responses of the S-RGOH sensor to 200 ppm NH_3 with prolonged NH_3 exposure time to 800 s. The saturated response of 16.1% was obtained at 800 s.

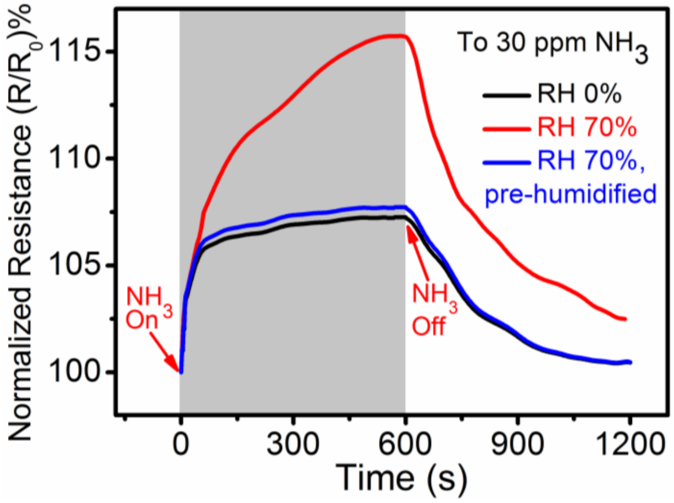

Figure S10. Responses of the S-RGOH sensor to 30 ppm NH_3 in the conditions of 0% RH (black), 70% RH without pre-humidification (red) and with pre-humidification (blue) treatment, respectively.

Calculation of the concentration of various saturated organic vapors:

The concentration of 10 mL methanol, ethanol, acetone, toluene and chloroform can be calculated according to the following formula:

$$C = (22.4QDP/VM) \times 10^9 \times (273+T_B)/(273+T_R) \quad \text{(Equation S3)}^{[5]}$$

where C is the concentration of the gas to be formulated (ppm), Q is the liquid volume (10 mL), D is the liquid density, P is the liquid purity, V is the chamber volume (1 L), M is the material molecular weight, T_B is the chamber temperature (RT) and T_R is the environment temperature (RT). As such, the detailed concentration is calculated as below:

Table S8. The translated concentrations of 10 mL organic vapors

Organic vapor	D (g/mL)	P	M (g/mol)	C (ppb)
10 mL methanol	0.79	100%	32	5.5
10 mL ethanol	0.79	100%	46	3.8
10 mL acetone	0.79	100%	58	3.0
10 mL toluene	0.87	100%	92	2.1
10 mL chloroform	1.49	100%	119	2.8

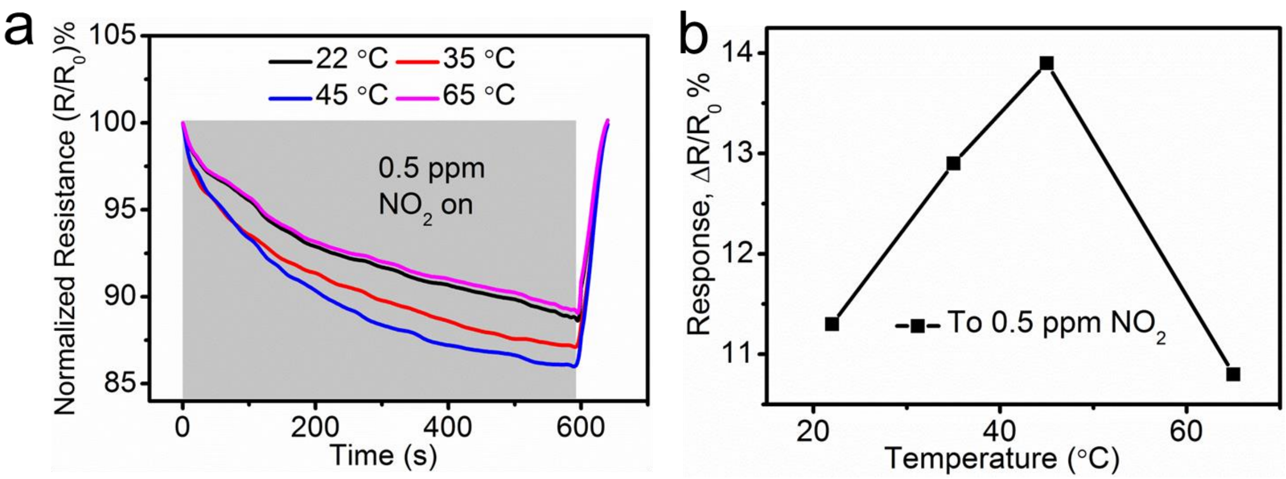

Figure S11. (a) Dynamic response of the S-RGOH sensor to 0.5 ppm NO_2 at 22, 35, 45 and 65 ℃ respectively. (b) Plot of the quantitative response of the sensor as a function of temperature.

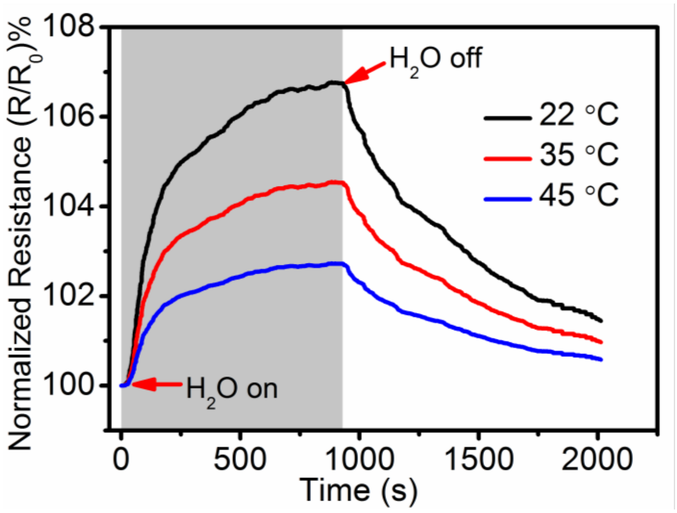

Figure S12. Dynamic responses of the S-RGOH sensor to 70% RH at the temperatures of 22, 35 and 45 ℃ respectively.

The characterization of microheater:

Because of the low thermal conductivity of the SiO_2 layer (~1 W/(m K)) and the relative high thermal conductivity of Si layer (~149 W/(m K)) on the Si/SiO_2 substrate,[9] the imbedded microheater can introduce a local heating effect to elevate the temperature of S-RGOH on the opposite side of substrate quickly with little power consumption and small device size. Importantly, the heat dissipation was fast after the direct current (DC) voltage is removed due to the local heating effect with small total heat generated. Thus, the temperature of the substrate can quickly return to the original state (< 51 s for 45 ℃).

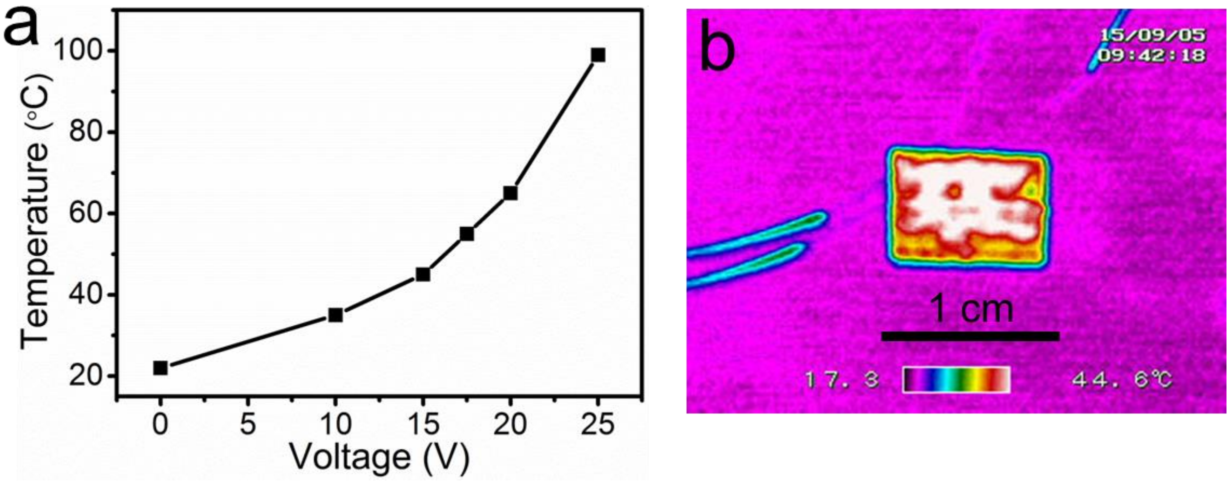

Figure S13. (a) Temperature of the substrate as a function of DC voltage applied to the microheater. (b) Optical image captured by an infrared camera showing the temperature of substrate was 45 ℃ when the DC voltage of 15 V was applied to the microheater.

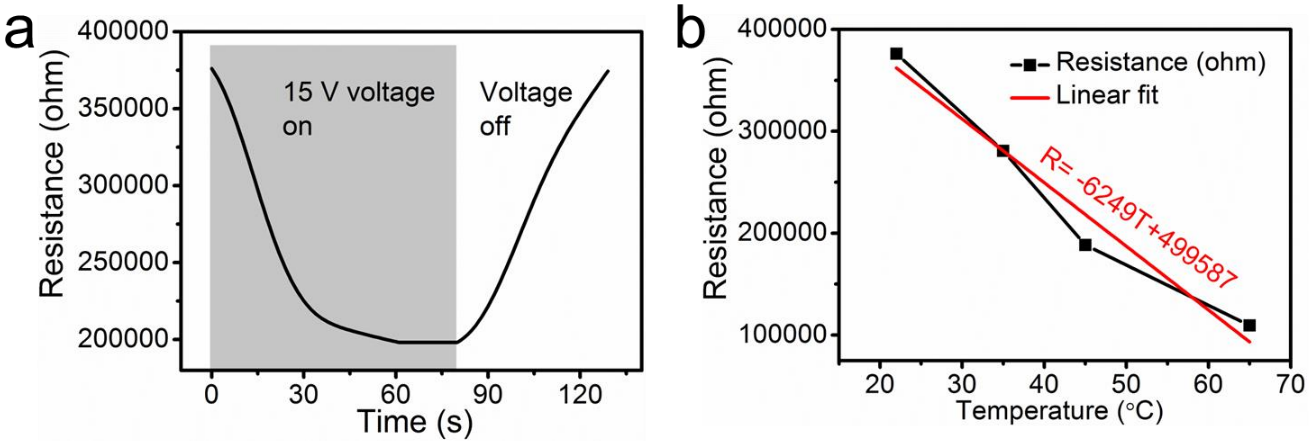

Figure S14. (a) Resistance change of the sensor as a function of time when the voltage of 15 V was applied to the microheater. The resistance decreased from 0 to 59 s after a 15 V DC voltage was applied on the microheater and then stabilized from 59 to 79 s. The resistance returned to the original state from 80 to 130 s when the DC voltage was turned off. It demonstrates the fast manipulation of the temperature of sensing materials by the microheater. (b) The measured (black dash-dot) and linearly fitted (red straight) resistance of the S-RGOH sensor as a function of temperature.

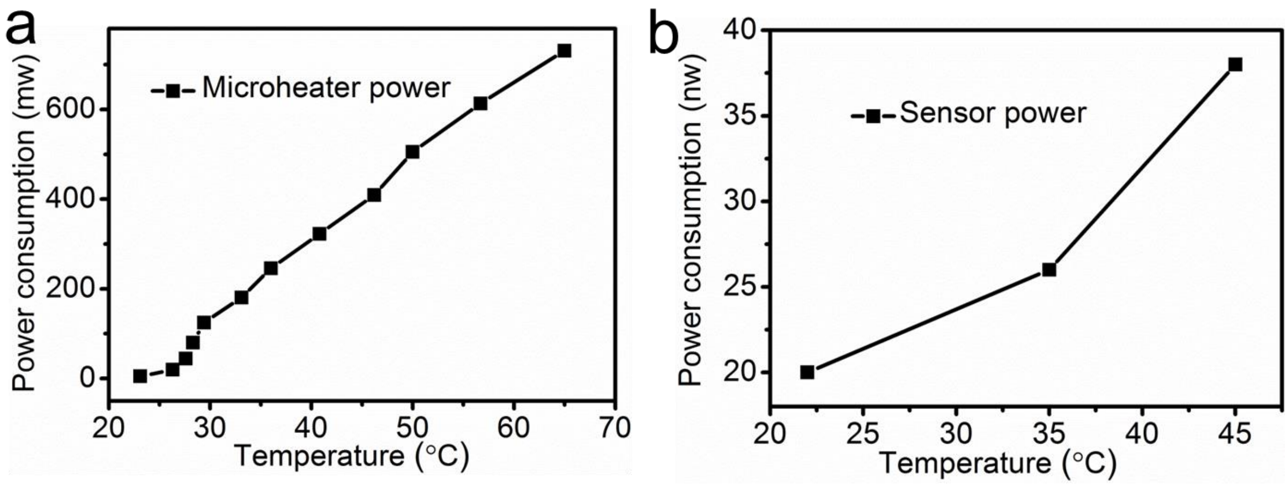

Figure S15. Power consumptions of the microheater (a) and gas sensor (b) respectively.

REFERENCES

[S1] L. T. Duy, D.-J. Kim, T. Q. Trung, V. Q. Dang, B.-Y. Kim, H. K. Moon, N.-E. Lee, *Adv. Funct. Mater.* **2015**, *25*, 883-890.

[S2] V. Dua, S. P. Surwade, S. Ammu, S. R. Agnihotra, S. Jain, K. E. Roberts, S. Park, R. S. Ruoff, S. K. Manohar, *Angew. Chem. Int. Ed.* **2010**, *49*, 2154-2157.

[S3] J. D. Fowler, M. J. Allen, V. C. Tung, Y. Yang, R. B. Kaner, B. H. Weiller, *ACS Nano* **2009**, *3*, 301-306.

[S4] G. Lu, L. E. Ocola, J. Chen, *Nanotechnology* **2009**, *20*, 445502.

[S5] L. Huang, Z. Wang, J. Zhang, J. Pu, Y. Lin, S. Xu, L. Shen, Q. Chen, W. Shi, *ACS Appl. Mater. Interfaces* **2014**, *6*, 7426-7433.

[S6] F. Yavari, Z. Chen, A. V. Thomas, W. Ren, H. M. Cheng, N. Koratkar, *Sci. Rep.* **2011**, *1*, 166.

[S7] L. Li, S. He, M. Liu, C. Zhang, W. Chen, *Anal. Chem.* **2015**, *87*, 1638-1645.

[S8] X. Zhou, X. Wang, B. Wang, Z. Chen, C. He, Y. Wu, *Sens. Actuators, B* **2014**, *193*, 340-348.

[S9] J. Wu, K. Tao, J. Miao, L. K. Norford, *ACS Appl. Mater. Interfaces* **2015**, *7*, 27502-27510.
